# Searching for psychosis: INTREPID (1): systems for detecting untreated and first-episode cases of psychosis in diverse settings

**DOI:** 10.1007/s00127-015-1013-6

**Published:** 2015-01-29

**Authors:** Craig Morgan, Maia Hibben, Oluyomi Esan, Sujit John, Vikram Patel, Helen A. Weiss, Robin M. Murray, Gerard Hutchinson, Oye Gureje, Rangaswamy Thara, Alex Cohen

**Affiliations:** 1 Health Service and Population Research Department, Centre for Epidemiology and Public Health, Institute of Psychiatry, Psychology & Neuroscience, King’s College London, London, UK; 2 National Institute for Health Research (NIHR) Mental Health Biomedical Research Centre at South London and Maudsley NHS Foundation Trust, King’s College London, London, UK; 3 Department of Psychiatry, University of the West Indies, Saint Augustine, Trinidad; 4 Department of Psychiatry, University of Ibadan, Ibadan, Nigeria; 5 Schizophrenia Research Foundation, Chennai, India; 6 Faculty of Epidemiology and Population Health, London School of Hygiene and Tropical Medicine, London, UK; 7 Psychosis Studies Department, Institute of Psychiatry, Psychology & Neuroscience, King’s College London, London, UK

**Keywords:** Psychoses, Case finding, India, Nigeria, Trinidad

## Abstract

**Purpose:**

Our understanding of psychotic disorders is largely based on studies conducted in North America, Europe and Australasia. Few methodologically robust and comparable studies have been carried out in other settings. INTREPID is a programme of research on psychoses in India, Nigeria, and Trinidad. As a platform for INTREPID, we sought to establish comprehensive systems for detecting representative samples of cases of psychosis by mapping and seeking to engage all professional and folk (traditional) providers and potential key informants in defined catchment areas.

**Method:**

We used a combination of official sources, local knowledge of principal investigators, and snowballing techniques.

**Results:**

The structure of the mental health systems in each catchment area was similar, but the content (i.e., type, extent, and nature) differed. Tunapuna–Piarco (Trinidad), for example, has the most comprehensive and accessible professional services. By contrast, Ibadan (Nigeria) has the most extensive folk (traditional) sector. We identified and engaged in our detection system—(a) all professional mental health services in each site (in- and outpatient services—Chengalpet, 6; Ibadan, 3; Trinidad, 5); (b) a wide range of folk providers (Chengalpet, 3 major healing sites; Ibadan, 19 healers; Trinidad: 12 healers); and c) a number of key informants, depending on need (Chengalpet, 361; Ibadan, 54; Trinidad, 1).

**Conclusions:**

Marked differences in mental health systems in each catchment area illustrate the necessity of developing tailored systems for the detection of representative samples of cases with untreated and first-episode psychosis as a basis for robust, comparative epidemiological studies.

**Electronic supplementary material:**

The online version of this article (doi:10.1007/s00127-015-1013-6) contains supplementary material, which is available to authorized users.

## Introduction

The overwhelming majority of research on schizophrenia and other psychoses is conducted in North America, Australasia and Europe [1]. The limited amount of research in other settings has tended to be methodologically limited and heterogeneous, making cross-country comparisons difficult [2, 3]. Notable exceptions are the WHO multi-country studies [4–6]. However, the questions raised by the WHO studies about the nature and determinants of variations in incidence and outcome globally have not been addressed in subsequent studies [3, 7]. Further, in the decades since the WHO studies were established there have been rapid and far reaching social and economic changes in many of the developing countries included in that programme (e.g., China, India, and Nigeria), with likely profound effects on the social epidemiology of mental disorders [8]. The INTREPID (India, Nigeria, Trinidad: Researching Psychosis in Diverse Settings) programme (Supplementary Appendix 1; see also: www.intrepidresearch.org) was established to—(a) develop robust, comparable methods for the study of schizophrenia and other psychoses in diverse settings (Phase 1); and (b) implement these in a multi-country study of the epidemiology, phenomenology, aetiology and outcome of psychoses (Phase 2). In this paper, our first from the programme, we report on the development of infrastructures within defined catchment areas in each participating country to identify representative samples of individuals with an untreated or first-episode psychosis—a critical first step in establishing robust epidemiological studies of psychoses that can provide insights into the impact of diverse social and cultural environments on the manifestations, occurrence, and outcome of these disorders, which in turn can inform further development of local systems of care [9].

### Searching for psychosis

Epidemiological studies of psychoses (i.e., of incidence rates, of risk factors, and of outcomes over time) require representative samples of untreated or first-episode cases. In line with a general trend in studies of psychoses (e.g., AESOP [10], EU-GEI [http://www.eu-gei.eu/]), for Phase 1 of INTREPID we have adopted broad case inclusion criteria (see Table 1). In North America, Australasia and Europe, samples of such cases are usually constructed by identifying individuals with psychosis who make contact for a first time with specialist mental health services [11, 12]. The assumption underlying this approach is that all those who develop a psychotic disorder will present to services, most within a short period following onset. In effect, specialist services provide a mechanism or infrastructure for the detection of representative samples of incident or untreated cases. However, this assumption is not tenable in settings where mental health services are relatively under-developed and/or under-used; periods of untreated psychosis among those who do present, moreover, are likely to be longer. A study in Chennai, India estimated that around 30 % of those with a psychotic disorder was never treated by mental health services [13]. A population-based study in rural Ethiopia found more than 90 % of those identified with a psychotic disorder had never been treated [14]. In such settings, reliance on specialist services to identify cases will produce biased samples; alternative strategies are needed.

**Table 1:** INTREPID case inclusion and exclusion criteria

Inclusion criteria
Age 18–64 years; resident in catchment area at time of case detection; evidence of psychotic symptoms or experiences in past 12 months; not treated with anti-psychotics for 3 continuous months prior to the start of recruitment
Exclusion criteria
Evidence of psychotic symptoms precipitated by an organic cause; central nervous system disease; transient psychotic symptoms resulting from acute intoxication

Despite this, much of the research on psychoses in low and middle income countries has been based on samples drawn from mental health services (e.g., [15–18]) (see also Table 2; [4, 5, 14, 18–30]). To our knowledge, there are no reports in the literature that systematically document, and subsequently seek to evaluate, methods for identifying cases of psychosis outside of mental health services in more than one setting. The WHO Determinants of Outcome of Severe Mental Disorders (DOSMeD) study did attempt to extend sampling to include traditional and spiritual healers and key informants. However, the details provided on this in published reports are slight, making evaluation and replication of the procedures difficult, and in only one (out of 4) of the developing country sites (Chandigarh, India) did the research team gain reasonable coverage of identified healers and informants. There is, moreover, no information on how many cases were identified through each source [5]. Some other studies have sought to incorporate providers and/or informants beyond the professional mental health care sector (e.g., in rural areas of India [31] and Botswana [32]). These, however, have tended to be small scale, covering small populations, and yielding fewer than 10 cases in each instance.

**Table 2:** Case finding methods used in studies of course and outcome of psychoses in low and middle income countries

Authors	Year	Location	Sample	Case finding methods^a^
Menezes et al.	1993	São Paulo, Brazil	*n* = 124 Any psychosis Age 15–44 years Prevalent cases	Hospital admissions Cases identified through three psychiatric hospitals and the psychiatric ward of the general hospital in the study catchment area in Sao Paulo, Brazil
Ran et al.	2001	Sichuan, China	*n* = 510 Schizophrenia No age criteria Prevalent cases	Community survey A cross-sectional survey of rural communities in the six townships of Xinjin County, China
Kebede et al.	2004	Butajira, Ethiopia	*n* = 318 Schizophrenia Age 15–49 years Prevalent cases	Community survey A two-stage community survey of rural communities in Butajira, Ethiopia. First-stage screen; second-stage assessment of screen positives and proportion of screen negatives
Kulhara et al.	1978	Chandigarh, India	*n* = 174 Schizophrenia Age15–60 years Incident cases	Hospital admissions and outpatient clinics Cases identified through the Department of Psychiatry, Postgraduate Institute of Medical Education and Research in Chandigarh, India
Kulhara et al.	1986	Chandigarh, India	*n* = 112 Schizophrenia Age 15–56 years Prevalent cases	Hospital admissions and outpatient clinics Members of the Department of Psychiatry, Postgraduate Institute of Medical Education and Research, were asked to refer patients with diagnosis of schizophrenia to the research team
Verghese et al.	1990	Multi-site study, India	*n* = 386 Schizophrenia (with duration of illness of less than 2 years) Age 15–45 years Incident cases	Outpatient clinics Consecutive patients who attended the psychiatry clinics of the participating centres
Thara et al.	1994	Madras Longitudinal Study, India	*n* = 90 Schizophrenia Age 15–45 years Incident cases	Hospital admissions or outpatient clinics Patients seen at the Department of Psychiatry, Government General Hospital, Madras, India
Padmavati et al.	1998	Chennai, India	*n* = 261 Any psychosis Mean age 36 years Prevalent cases	Community Survey Door-to-door survey of two residential areas with a population of around 100,000
Murthy et al.	2005	Rural Karnataka, India	*n* = 100 Schizophrenia Age: not specified Prevalent cases	Outpatient clinics Patients attending eight outreach clinics and who were drug naive or had discontinued treatment after initial contact and had not received antipsychotic treatment for the previous 6 months
Kurihara et al.	2000	Bali, Indonesia	*n* = 59 Schizophrenia Mean age 27 years (unclear if this is at baseline or 5-year follow-up) Prevalent cases	Hospital admissions Consecutive patients with no prior admissions admitted to Bangli State Mental Hospital
Hickling et al.	1995	Jamaica	*n* = 317 Non-affective psychoses Age 15–54 years Incident cases	Outpatient clinics and community services All patients presenting to mental health services for the first time
Makanjoula et al.	1987	Ilesa, NIgeria	*n* = 116 Schizophreniform Age: not specified Prevalent cases	Hospital admissions Consecutive new patients presenting to a psychiatric unit
Oosthuizen et al.	2005	Cape Town, South Africa	*n* = 57 Non-affective psychoses Age 16–55 years Incident cases	Hospital admissions Individuals with a first-episode psychosis presenting to the Stikland–Tygerberg Hospital
Bhugra et al.	1996	Trinidad	*n* = 56 Any psychosis Age 15–54 years Incident cases	Hospital admissions, outpatient clinics and community services All patients with a possible psychosis presenting to mental health services (including prison in-reach service, the private sector, and mental health officers) for a first time
Ganev et al.	1998	Sofia, Bulgaria	*n* = 60 Any psychosis Age 16–45 years Prevalent cases	Hospital admissions and outpatient clinics Cases with an onset of illness of less than 2 years at the time of assessment
Hopper et al.	2007	China	*n* = 89 Schizophrenia Mean age 42 years Prevalent cases	Community survey Persons living in 8 defined urban catchment areas and diagnosed with schizophrenia in the first national epidemiological survey of mental disorders in 1982
Hopper et al.	2007	Cali, Colombia (WHO IPSS Study)	*n* = 101 Schizophrenia Age 15–45 years Mainly incident cases	Hospital admissions Inpatients San Isidro Psychiatric Hospital; 90 % was first episode
Hopper et al.	2007	Agra, India (WHO IPSS Study)	*n* = 140 Schizophrenia Age 15–45 years Prevalent cases	Outpatient clinics Patients attending the outpatient department of Agra Mental Hospital
Jablensky et al. (WHO Determinants of Outcome of Severe Mental Disorders Study)	1992	Multi-Country	*n* = 586 from developing countries (Agra, Cali Chandigarh, Ibadan) Any psychosis Age 15–54 years Incident cases	All mental health services, healers and informants In addition, mental health facilities outside the study catchment areas were monitored; leakage studies were conducted. Details of screening of healers and informants are limited (a) Centres that applied the case finding without modification: Aarhus (Denmark), Chandigarh (India), Dublin (Ireland), Honolulu (Hawaii), Moscow (Soviet Union), Nagasaki (Japan) and Nottingham (UK) (b) Centres that had to introduce modifications: Agra (India); Cali (Colombia); Ibadan (Nigeria); Prague (Czechoslovakia); Rochester (USA)

Incident cases refer to samples of first episode or first contact cases

^a^We provide as much details as we could glean from published reports

### Health care systems

Kleinman’s model of health care systems provides a useful framework for formalising approaches to identifying cases of psychosis across diverse settings[33]. In this model, three distinct but overlapping sectors in which illness is understood and managed constitute the near universal structure of health care systems—the professional (i.e., medical establishment), folk (i.e., spiritual and traditional healers), and popular (i.e., informal efforts to manage illness, e.g., self medication, advice from friends and family, etc.).

Cases of psychosis (as for all illnesses and disorders) initially arise and are managed within the popular sector; the extent of and permeability of the boundaries between the popular, the folk, and professional sectors will influence how many cases become visible in these sectors, i.e., how many can be detected. The more permeable the boundaries between the professional and the other sectors, then, the more cases will appear in (and thereby be detectable by screening) professional mental health services. This is the basis of the assumption already noted underlying studies of incidence and aetiology in high income countries, i.e., all cases will eventually breach the boundaries and present to professional services. Where this assumption fails, efforts to identify cases have to extend into the folk and popular sectors.

### Aim

As a platform for INTREPID, we sought to establish comprehensive systems for detecting cases of psychosis by mapping and seeking to engage (i.e., secure agreement to identify and refer potential cases) all professional and folk providers and potential key informants (as a window into the popular sector) in defined catchment areas in our study settings—Chengalpet taluk, near Chennai, India; Ona Ara and Ibadan South East, Ibadan, Nigeria; and Tunapuna–Piarco, Trinidad and Tobago. In presenting findings on the content of the health care systems in each catchment area and on the establishment of detection systems, we: (a) provide uniquely detailed snapshots of mental health care systems in three diverse settings; and (b) provide a methodological template that can be adapted as a basis for the generation of representative samples psychosis in other settings.

## Methods

**Fig. 1:**
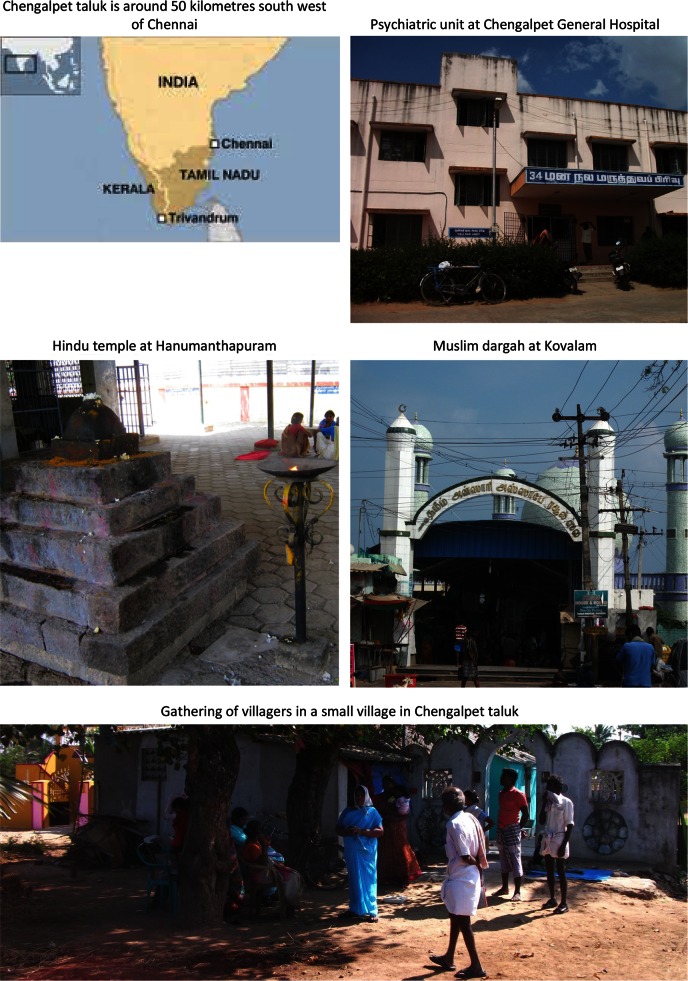
Chengalpet taluk, Tamil Nadu, India

**Fig. 2:**
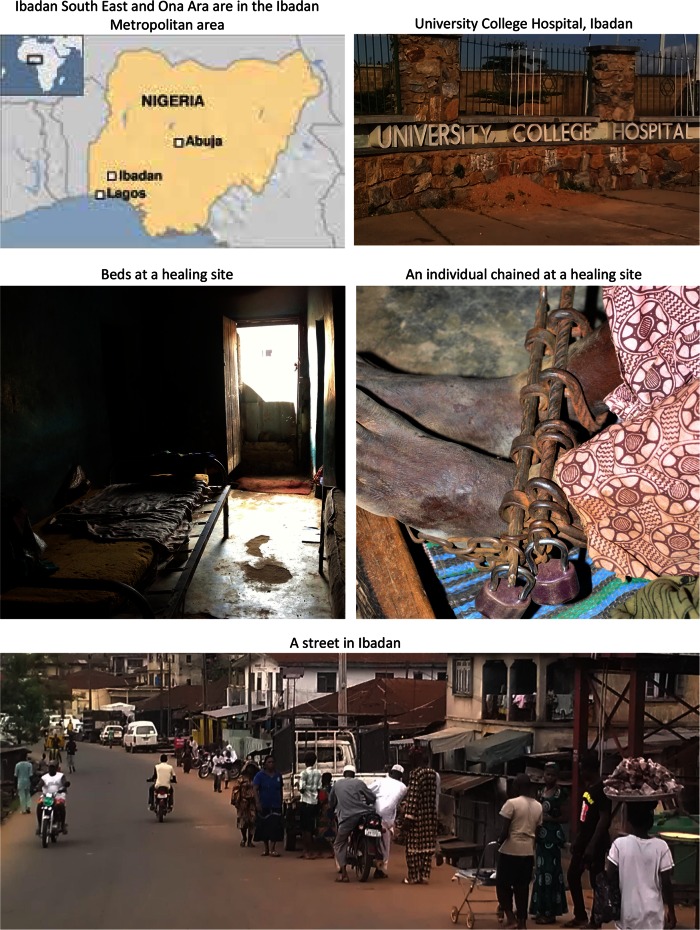
Ibadan South East and Ona Ara, Ibadan, Nigeria

**Fig. 3:**
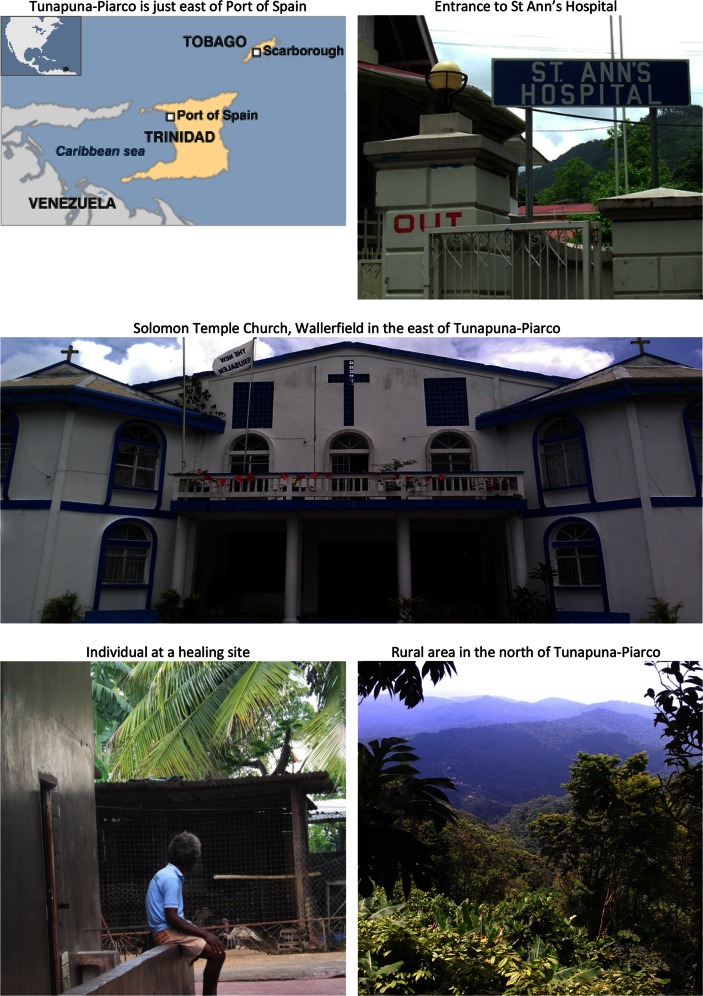
Tunapuna–Piarco, Trinidad and Tobago

The sites included in INTREPID are economically, socially and culturally diverse and are all located in countries that have undergone major changes in recent decades, including rapid increases in urban populations and unprecedented economic growth (Tables 3, 4). Supplementary Appendix 2 provides summary descriptions of each site, and Figs. 1, 2, and 3 contains maps and illustrative photos from each site.

**Table 3:** Economic, development and health indicators

	India		Nigeria		Trinidad and Tobago
	National	Tamil Nadu^†^	National	Oyo State^††^	National^†††^
Urban population (a, b) (2011)	31.2 % (3.8 % increase since 2001)	48.5 % (4.4 % increase since 2001)	49.6 % (5.7 % increase since 2001)	No data^^^	13.7 % (2.9 % increase since 2001)
Projected annual rate of urbanisation (a) (2010–2015, estimated)	2.4 %	No data**	3.8 %	No data^^^	2.9 %
Economic (GDP) growth (d)					
2006–2010 (average per year)	7.1 %	9.5 %	4.1 %	No data	3.0 %
2012	3.2 %	9.4 %	6.5 %	No data	1.5 %
Poverty (c) (i.e., living on less than $1.25 per day) (2010)	32.7 %	32.4 %	68.0 %	No data	4.0 %
Income Gini‡ coefficient (c) (2010)	33.4	No data	48.8	No data	No data
Human Development Index (c) (2012)	0.55	0.74	0.47	No data	0.76
Infant mortality per 1,000 live births (c) (2010)	47	22	78	22	25
Life expectancy at birth (years) (c) (2012)	65.8	72.4	52.3	No data	70.3
Literacy, 15 + years (c, e, f) (2010–2011)					
Men	82.1 %	86.9 %	72.1 %	76.3 %	99.2 %
Women	65.5 %	73.9 %	50.4 %	65.8 %	98.5 %

^‡^The income Gini coefficient is a measure of inequality

^†^Our catchment area in India (Chengalpet taluk) is in Tamil Nadu state

^††^Our catchment area in Nigeria (Ibadan South East and Ona Ara) is in Oyo state

^†††^There are no data available for regions in Trinidad and Tobago

** Information on projected rate of urbanisation not available. Between the 1991 and 2011 census, the urban population in Tamil Nadu grew by 14.3 % (from 34.2 to 48.5 %)

^^^Directly comparable information on urban populations in Oyo State not available. Ibadan is the third largest city in Nigeria (after Lagos and Kano), with (at 2006 census) a population of around 2,338,659

(a) World Urbanisation Prospectus, 2011 Revision. United Nations (http://esa.un.org/unpd/wup/CD-ROM/Urban–Rural-Population.htm)

(b) 2011 Indian Census

(c) Human Development Report 2013

(d) The World Bank (http://data.worldbank.org/indicator/NY.GDP.MKTP.KD.ZG)

(e) Report of the National Literacy Survey, 2010. Abuja, Nigeria: National Bureau of Statistics (http://resourcedat.com/wp-content/uploads/2012/04/National-Literacy-Survey-2010.pdf.)

(f) The World Factbook; 2013. CIA (https://www.cia.gov/library/publications/the-world-factbook/fields/2103.html)

**Table 4:** Population estimates for study catchment areas

	Chengalpet (a) India 2011	Ibadan (b) Nigeria 2006		Tunapuna–Piarco (c) Trinidad 2011
		Ibadan South East	Ona Ara	
Total population	412,289	266,457	265,571	248,656
Men	209,310	130,334	130,615	123,232
Women	202,979	136,123	134,956	125,424
Population aged 18–65 years*	259,742^†^	152,404**	148,852**	169,042
Men	131,865^†^	73,046**	71,443**	84,239
Women	127,877^†^	79,358**	77,409**	84,803

* The age range for cases we will seek to identify during case recruitment is 18–65 years

^†^Population aged 18–65 estimated using proportions in this age group for Kancheepuram District (i.e., 63 % aged 18–65; same for men and women)

** Population aged 15–64

(a) Census of India. Provisional Population Totals, Tamil Nadu-Census 2011 Sub District (Taluk) Level. [cited 2013 25 August]; Available from: http://www.census.tn.nic.in/census2011data/PPT_taluk_data_final.pdf

(b) National Population Commission of Nigeria. Population Distribution by Sex, State, LGA & Senatorial District. Abuja, Nigeria: National Population Commission of Nigeria; 2010

(c) Central Statistical Office, Ministry of Planning and Sustainable Development. Trinidad and Tobago 2011 Population and Housing Census Demographic Report. Port of Spain, Trinidad: Government of Trinidad and Tobago; 2012

### Identifying providers and informants

In each of our study catchment areas, we sought to identify all providers in the professional and folk sectors and, as a means of penetrating the popular sector, potential key informants who have local knowledge of individuals in their communities with psychosis. Our methods to achieve this had, inevitably, to be flexible and tailored to each setting. The inclusion and exclusion criteria we have applied in the next stage to define untreated and first-episode cases are set out in Table 1.

As a starting point, in each site we drew from the following to create an initial list of providers and informants

1. Local health and government administrative departments and officials. Sources used included—Chengalpet, a database listing all registered medical and hospital facilities in the district; Ibadan, a list of all public and private health care facilities; Tunapuna–Piarco, the Ministry of Health website (http://www.health.gov.tt/) and a list of public facilities provided by the regional hospital.
2. Local knowledge of site principal investigators and researchers, including networks of contacts. Contacts used included—Chengalpet, coordinator of rural mental health programmes at the Schizophrenia Research Foundation; Ibadan, Chair of State Board of Traditional Medicine; Tunapuna–Piarco, leaders of major temples, shrines, and churches.
3. Information on pathways to care from a sample of patients with psychosis and families already in contact with local mental health services (Chengalpet *n* = 62; Ibadan *n* = 20; Tunapuna–Piarco *n* = 35), collected using the pathways section of the WHO Personal and Psychiatric History Schedule (1993).

We next used snowballing techniques. Using the information gained from the sources listed above, we asked each identified practitioner, healer, and informant whether they knew of others who provided similar services or who had relevant local knowledge.

### Engagement

Following identification, we approached each provider and potential informant to seek their involvement in actively identifying and referring cases who potentially met our inclusion criteria. To facilitate this, we conducted a series of in-depth interviews and focus groups in each site with providers, informants, and relatives of those with psychoses to establish local understandings of psychosis (i.e., terms used, typical signs, causes, usual responses, etc.) (in preparation). These both enabled us to approach providers and informants using local terms to explain the purpose and nature of the project and formed the basis for training and information materials, using local idioms, that we produced to ensure a shared understanding of the types of experiences and behaviours we were interested in.

### Information collated

Using a specifically designed proforma, we collected the following information (as appropriate) on each provider and informant, from both publicly available documentary sources and the providers and informants directly—name, location, service(s) provided, staff, types and numbers of patients seen (including whether any with psychosis), whether public (state funded or subsidised) or private, and costs.

## Findings

In each catchment area, we identified a wide array of services and providers in the professional and folk sectors and a range of key informants. From this, we constructed an initial set of services, providers and key informants to monitor and screen for untreated and first-episode cases of psychosis (Table 5; Supplementary Tables 1–3), i.e., a detection system.

**Table 5:** Overview of providers, healers and key informants identified by site

	Chennai, India (see supplementary Table 1 for detail)						Ibadan, Nigeria (see supplementary Table 2 for detail)						Trinidad (see supplementary Table 3 for detail)					
	In catchment area			Outside catchment area			In catchment area			Outside catchment area			In catchment area			Outside catchment area		
	No.^a^	Beds	Costs^b^	No.^a^	Beds	Costs^b^	No.^a^	Beds	Costs^b^	No.^a^	Beds	Costs^b^	No.^a^	Beds	Costs^b^	No.^a^	Beds	Costs^b^
Professional sector																		
Public	2 (2)	10	None	4 (0)	1882	Nominal	0 (–)	0	–	2 (2)	88	$70^c^	4 (4)	0^i^	None	1 (1)	958	None
NGO	1 (1)	12	None	2 (1)	150	$0–$40	0 (–)	0	–	0 (–)	0	–	0 (–)	0	–	0 (–)	0	–
Private	4 (4)	33	$5–$480	123 (2)^f^	100^g^	$16–$399	0 (–)	0	–	1 (1)	36	[?]	1 (1)	0^i^	$62–$109	0 (–)	0	–
Folk sector																		
Healing sites, healers	3 (3)^d^	18–22^e^	$16–$1610	–	–	–	19 (19)	200	$31–$737^h^	–	–	–	23 (12)	0	Unknown	–	–	–
Popular sector																		
Key informants	619 (361)	–	–	–	–	–	54 (54)	–	–	–	–	–	1 (1)	–	–	–	–	–

^a^Number of providers; the numbers being monitored to identify cases during case recruitment are in brackets

^b^Costs are estimated in US dollars for comparison and are per week, unless specified

^c^These are average costs after payment of an initial deposit (around 190 US dollars) that covers the first few weeks of care

^d^We identified three healing sites (a Hindu temple, a Muslim dargah and a Pentecostal Church). At the temple and dargah, a number of individual healers provided services in and around the sites. (See Supplementary Table 1.)

^e^The temple and dargah have provision for individuals to stay at their sites for the duration of the healing

^f^In Chennai city, there are 3 private hospitals and around 120 private psychiatric practices. We identified two private practices that specialised in the treatment of those with psychotic disorders

^g^This does not include provision for private psychiatrists to admit patients to general hospital beds

^h^Total costs (rather than weekly)

^i^Patients can be admitted to general hospital beds (see supplementary Table 3)

### Chengalpet, India

#### Professional sector

Professional mental health care within Chengalpet taluk comprises public (state funded), charitable non-governmental organisation (NGO), and private services.

There are four hospitals or residential facilities (one public, one NGO run, two private) that have psychiatric units and admit individuals with psychotic disorders, with a total of 55 beds (10 public; 12 NGO; 33 private). The public hospital (Chengalpet General Hospital (GH)) provides services free of charge, but a family member is required to stay with each patient during their stay. Outpatient appointments are offered on discharge from the public and private hospitals, but there is no formal system for tracking patients who do not attend. The NGO, Banyan,  holds 5 outpatient clinics per month at its facility, at which an average of around 50 patients are seen, most of whom have a serious mental disorder.

In addition to hospital and residential-based care, there are some community-based services. Across India, the state funded District Mental Health Programme (DMHP) provides a psychiatrist, a psychologist and a social worker for each district. This team conducts weekly outreach outpatient clinics in local hospitals and health clinics. Each week, around 300 patients are seen in DMHP clinics in Chengalpet taluk. Further services are provided by the Schizophrenia Research Foundation (SCARF), a NGO based on Chennai that provides a range of mental health services for those with schizophrenia.

Public and NGO services are free; private hospitals charge up to 30,000 rupees (around 480 US dollars) per week for admission, assessments and medication, and private psychiatrists charge around 300 rupees (around 5 US dollars) for consultations and for medication per month.

#### Help-seeking out of area

In Chengalpet, health services are not catchment area based and some patients and their families inevitably seek care outside of the taluk. For example, the close proximity of Chennai means services within the city are accessible. We consequently extended our mapping of professional mental health services and providers into Chennai. In the city, inpatient care is provided by—(a) a specialist psychiatric hospital, the Institute of Mental Health, which serves the populations of Tamil Nadu and Pondicherry and is the second largest psychiatric hospital in India (1,800 beds); (b) psychiatric units in three public hospitals (82 beds in total); (c) SCARF (150 beds); and (d) psychiatric units in three private medical colleges (i.e., training institutions with attached teaching hospitals) (100 beds in total).

The public, NGO and private facilities noted above also provide outpatient and rehabilitation services. In addition, Banyan provides community-based services (around 300 registered patients with psychosis). Further, there are around 120 psychiatrists who operate private practices. Of these, two were identified through our pathways data as providing care particularly for those with a psychotic disorder.

#### Folk sector

Folk sector provision for those with mental health problems in Chengalpet is largely faith based and delivered by religious practitioners and healers located within churches or temples. This posed particular difficulties in developing a comprehensive map of such provision, as there are numerous small scale churches and congregations throughout Chengalpet. As a consequence, we were restricted to documenting the more prominent places of worship and healing.

There are two large religious healing centres in Chengalpet taluk. One, a Hindu temple at Hanumanthapuram (a small village around 18 kilometres from Chengalpet town), is well known within the region as a treatment centre for those with a mental disorder. The interventions involve the sufferer (and sometimes family) staying in or around the temple for around 40 days and undergoing ritualised ceremonies. At any point, 8–10 individuals are resident in or near the temple. There are no charges. The other, a Muslim dargah at Kovalam (around 40 kilometres from Chengalpet town), is again well known as a place of healing for those with a mental disorder. The interventions involve prayers and rituals. At any point, 12–15 individuals are resident at the dargah to receive treatment. Costs of consultation range from 10,000 to 100,000 rupees (around 160–1,610 US dollars). The leaders of both identified other faith healers associated with their centres: approximately 10–12 magico-religious practitioners operate around the Hindu temple and approximately 12–15 Muslim faith healers operate around the dargah. However, a majority of these practitioners and healers were guarded when approached and could not be engaged.

In addition to healers operating within the two main religions of the region, there are a large number of Christian churches (approximately 200) of various denominations. In line with our approach described above, we did identify six of the larger churches. Of these, in only one Pentecostal church did the pastor say specific provisions were made for members of the congregation with mental health problems.

#### Key informants

We identified and engaged (i.e., secured agreement to identify potential cases) a range of key informants in prominent local positions with a good knowledge of village communities within Chengalpet—342 Balwadis (pre-school teachers;); 18 village health nurses (each covering approximately 10–12 villages within Chengalpet); 27 primary care nurses; 17 primary care physicians; around 100 members of the Panchayats (village assemblies); the local police; and 15 religious leaders.

### Ibadan South East and Ona Ara, Ibadan, Nigeria

#### Professional sector

Professional mental health care available to residents of Ibadan South East and Ona Ara is limited and there are no services located within the boundaries of the catchment area. There are, however, two public hospitals that have psychiatric units and admit individuals with psychotic disorders in neighbouring areas that serve all of Ibadan and surrounding districts—(a) Adeoyo State Hospital, a general hospital with a 16 bed psychiatric unit (located in Ibadan South West); and (b) University College Hospital (UCH), a central government owned hospital with a 62 bed psychiatric unit (located in Ibadan North). The psychiatric units at both hospitals provide inpatient and outpatient care. In addition to these public facilities, there is a 36 bed private psychiatric hospital (New World Specialist Hospital, located in Ibadan South West) that is accessible to residents of Ibadan South East and Ona Ara. The New World hospital provides both in- and outpatient services. No services are free. UCH, for example, requires a deposit of 30,000 naira (around 185 US dollars) prior to admission and charges 1,600 naira per night (around 10 US dollars).

#### Folk sector

There is a large and diverse folk sector in Ibadan comprising healers and practitioners working within various traditions. The scale of the folk sector meant we were restricted, as in Chennai, to identifying the more prominent healers and practitioners.

Initially, then, we identified 19 healing sites and healers (12 spiritual, 7 traditional). Broadly, spiritual healers rely primarily on faith-based rituals (e.g., prayer, fasting, and laying on of hands) and traditional healers incorporate use of herbs, roots and sacrifices. Individuals receiving treatment are usually resident with healers for up to 6 months. Costs vary greatly and can be as high as 120,000 naira (approximately 737 US dollars) and chaining is common (Fig. 2). We were able to engage with all 19 identified.

#### Key informants

While there are no specialist mental health services within Ibadan South East and Ona Ara, there are a large number of government funded primary health centres [PHC] that provide general health care. Individuals with a mental disorder occasionally present to these clinics and staff have good local knowledge of the areas served. Within our catchment area, there are 17 PHCs and staff at all were engaged as key informants. Similarly, in the area there are a large number of relatively small scale private clinics that provide a range of specialist services (e.g., gynaecology, obstetrics, etc.) and we engaged staff at 33 of these clinics as key informants (i.e., secured agreement to identify potential cases). In addition, the spiritual and traditional healers we identified in effect doubled as key informants, as their positions within their communities mean they are able to identify individuals with a serious mental disorder beyond those they treat.

### Tunapuna–Piarco, Trinidad

#### Professional Sector

Professional mental health care within Tunapuna–Piarco comprises public and private services. There are no NGO run mental health services. Public mental health services in Trinidad closely resemble the model common in western Europe (i.e., public provision of free catchment area-based in- and outpatient services with relatively few private services).

The main public psychiatric hospital in Trinidad is St Ann’s Hospital located in Port of Spain, which has 27 wards, around 1,000 beds, and employs 35 medical staff. Although not in Tunapuna–Piarco, patients from that area requiring inpatient care are usually admitted to St Ann’s and then followed on discharge at outpatient clinics run by the Tunapuna–Piarco mental health team (see below). The main general hospital in Tunapuna–Piarco (the Eric Williams Medical Sciences Complex (EWMSC)) does not have dedicated psychiatric beds, but does admit patients with a mental disorder to general wards.

The Tunapuna–Piarco area mental health team holds outpatient clinics in two primary health facilities—weekly at Ticaragua Health Centre (150–200 active patients at any point) and fortnightly at Arima District Health Centre (80–120 active patients at any point). Those living in the rural parts of the catchment area will often be seen at home or will visit their local health clinic where a District Health Visitor can administer depot injections. The EWMSC outpatient clinic operates twice weekly and sees approximately 50 persons per week. Further, Trinidad has a publicly funded prison psychiatric in-reach service that provides care for prisoners in four prisons, all located in Arouca, in the eastern part of Tunapuna–Piarco.

There is one private hospital in Tunapuna–Piarco (St Augustine) that provides mental health care. There are no dedicated psychiatric beds in the facility; however, a psychiatric patient can be admitted at a cost of 2,000 Trinidad and Tobago dollars (approximately 312 US dollars) per day. There is no dedicated outpatient facility in St Augustine Private Hospital; patients are seen on a private appointment only basis (costs range from 400 to 700 Trinidad and Tobago dollars (approximately 62–109 US dollars) per session).

#### Folk sector

Trinidad is culturally and religiously diverse. Reflecting this, we identified 24 traditional and spiritual healers within Tunapuna–Piarco. Most practised within the framework of one of the many religious denominations or churches present in Trinidad (e.g., Hindu, Muslim, Baptist, Pentecostal, and Catholic) and many were pastors or church leaders, in effect offering services to members of their congregations. Many provide healing for individuals suffering from a mental disorder or from spiritually or religiously framed problems (e.g., demonic possession, victims of witchcraft or obeah), and the interventions ranged from prayers and cleansing rituals to exorcisms and spirit removals. Unlike in Chennai and Ibadan, however, there were no healers who specialised in the treatment of mental disorder and there were no large scale healing centres. In other words, the folk sector was more diverse and disparate, with limited evidence of specialisation, the consequence being that it is yet more difficult to fully map provision within this catchment area. Of the 24 healers identified, we were able to engage with 12.

#### Key informants

District health visitors (DHV) provide community health care to people in their homes in Trinidad. So far as these professionals have good connections within, and knowledge of, local communities (especially the more rural and less densely populated regions), they are potential key informants and we engaged the one DHV working in Tunapuna–Piarco, who agreed to identify potential cases. As in Ibadan, healers also doubled as key informants for their local communities in Tunapuna–Piarco.

### Finding psychosis

From the providers and informants identified, we derived a network of contacts in each site to constitute an initial system for detecting untreated cases (Table 4; Supplementary Tables 1, 2, 3). The inclusion of providers and informants, from those identified, for initial regular monitoring was dependent on their willingness to engage and, for providers, on confirmation that individuals with a (possible) psychotic disorder were seen or treated.

## Discussion

As far as we are aware, this is the first systematic attempt to map and engage providers and informants in multiple settings as a basis for rigorous, comparative epidemiological studies of psychosis. In doing this, we established a functioning system for detecting cases that we have subsequently piloted (in preparation). As expected from Kleinman’s model, the overall structure of the local health care system was similar in all sites (i.e., in each there were identifiable professional, folk and popular sectors within which episodes of mental disorder are managed), but the content (i.e., type, extent and nature of provision) differed.[33] In Trinidad, for example, professional mental health services are more comprehensive than in India and Nigeria; in our catchment area in Ibadan the only available services are out of area hospitals, which are expensive. Not surprisingly, given this, the folk sector in Ibadan comprises a relatively large number of healers specialising in providing interventions for those who suffer from mental disorders; such specialisation was evident to some extent in Chengalpet, but not in Tunapuna–Piarco. In all sites, healing practices within the folk sector reflected the diversity of local cultural and religious groups, practices and beliefs. This illustrates the necessity, in constructing cross-culturally comparable, representative samples of untreated and first-episode psychosis, of developing tailored detection systems that take into consideration the structure and content of local health care systems. What is unique in this report is the detail we provide and the consequent transparency in making explicit the extent of, and limitations to, our coverage of each sector. As such, it provides a methodological template that can be adapted to other settings.

### Limitations

There are two primary limitations to the procedures and findings reported in this paper.

First, the extent to which we were able to comprehensively map all providers and to identify a sufficient number of key informants in each site is unclear. On the basis of lists from government agencies and the local knowledge of study principal investigators, all of whom work within the professional sector within the catchment areas, we are confident that we identified all mental health services. We cannot be as confident about providers within the folk sector and key informants. The diversity of provision within the folk sectors in each site is striking and this presents considerable challenges, especially when, as in Trinidad, healing is non-specialised and small scale. The process of identifying providers, therefore, has to be iterative and ongoing, and this emphasises the importance of developing infrastructures for programmes of research that are conducted over relatively long time periods. This is our aim with INTREPID and following the initial intensive mapping stage we will continue to identify providers and informants during subsequent stages.

Second, we were not able to engage all providers in the study. The reasons for this vary from site to site, but there were common themes. For example, healers were often guarded. For some, the concern was that, in referring cases to us, we would seek to take over their care, thereby depriving healers of income. For others, a more general suspicion regarding the intentions of the research was evident. In this respect, it is important that research is presented to providers and potential informants in such a way that is both clear and acknowledges likely concerns. What this further indicates is the value of developing trust, which takes time, once again pointing to engagement as an iterative process and the necessity of establishing long-term projects, such as INTREPID, in which there is the space to develop relationships with providers across all sectors.

It is more accurate, then, to consider the infrastructure established in Phase I of INTREPID as work in progress. As the programme continues and further providers and informants are identified and engaged, its scope will expand and deepen. This noted, as far as we are aware, the coverage of even these initial detection systems is much greater than in any previous programme.

### Expanding the horizons of psychosis research

Conducting research on psychosis in settings beyond North America, Europe and Australasia is important for at least two reasons. First, from a research perspective, it will greatly enhance our understanding of all facets of psychosis—epidemiology, phenomenology, aetiology, course and outcome. Second, from a public health perspective, it will provide invaluable information on the nature and extent of need, and on service provision, in diverse settings [7].

That research in more countries which has the potential to achieve both of these is already clear from some of the more robust studies that have been conducted to date. For example, there are intriguing hints that the social distribution of psychoses may differ from that commonly found in Europe (e.g., low rates in women [14]; low rates in urban areas [16]). These variations may provide opportunities to investigate putative risk factors, especially in settings undergoing rapid social and economic changes. Further, the WHO multi-country studies raised significant questions about the nature and determinants of variations in course and outcome, particularly the role of social and cultural contexts, but these have not since been investigated in subsequent robust multi-country studies. Taking up these and other questions will be at the centre of Phase 2 of INTREPID and the work described here seeks to lay the foundations for this and for other studies in more diverse settings.

## Electronic supplementary material

Below is the link to the electronic supplementary material.

Supplementary material 1 (DOCX 59 kb)
