# Supplementary material for: Searching for psychosis: INTREPID (1): systems for detecting untreated and first-episode cases of psychosis in diverse settings
Source: Soc Psychiatry Psychiatr Epidemiol. 2015 Jan 29;50(6):879–93. doi: 10.1007/s00127-015-1013-6 (PMC4441747; doi:10.1007/s00127-015-1013-6)
Supplement: Supplementary file 1 — Supplementary material 1 (DOCX 59 kb) [file 127_2015_1013_MOESM1_ESM.docx]

**Appendix 1.** Overview of INTREPID.

INTREPID is a programme of research designed to: a) develop robust, comparable methods for the study of schizophrenia and other psychoses in diverse settings (Phase 1); and b) implement these in a multi-country study of the epidemiology, phenomenology, aetiology and outcome of psychoses (Phase 2). Phase 1 of INTREPID is being conducted in defined catchment areas in three countries: Chengalpet talk (near Chennai), India; Ibadan South East and Ona Ara, Ibadan, Nigeria; and Tunapuna-Piarco, Trinidad.

**Aims**

Our overall aims for Phase 1, in a series of pilot and feasibility studies, are:

1. To implement and evaluate strategies for: a) identifying and recruiting untreated and first-episode cases of psychosis; b) identifying and recruiting representative controls; and c) following cases over time, while minimising attrition
2. To translate, validate (where necessary) and test the feasibility of a core set of instruments and procedures to collect comparable information across diverse settings on psychopathology, biological and social (including social and cultural contexts) exposures, and outcome

To meet these aims, Phase 1 comprises three pilot studies and evaluations of procedures for assessing cross-culturally psychopathology, putative risk factors and course and outcome. Brief details of the aims of each component are provided here.

**Pilot Study (1)**

**Aim** To implement and evaluate a multi-pronged strategy for identifying and recruiting incident (defined at this stage as untreated and first episode) cases of psychosis

*Inclusion Criteria*

Age 18-64 years; resident in catchment area at time of case detection; evidence of psychotic symptoms or experiences in past 12 months; not treated with anti-psychotics for 3 continuous months prior to the start of recruitment

*Exclusion Criteria*

Evidence of psychotic symptoms precipitated by an organic cause; central nervous system disease; transient psychotic symptoms resulting from acute intoxication.

This pilot has three components:

a) Mapping of providers, healers and key informants within each catchment area to establish a system for detecting untreated and first episode cases of psychosis

b) Conducting focus groups and in-depth interviews with providers, relatives and key informants to gather information on local understandings of serious mental disorder, as a basis for producing information materials framed in local terms on the types of individuals we are looking for

c) Implementing and evaluating case finding over a 6 month period in each programme catchment area

**Pilot Study (2)**

**Aim** To implement and evaluate a neighbourhood-based strategy for identifying and recruiting representative controls

**Pilot Study (3)**

**Aim** To implement and evaluate strategies for following cases and controls at 12 months, while minimising attrition

**Instruments and Procedures**

**Aim** To translate, validate (where necessary) and test the feasibility of a core set of instruments (see Table)

**Table.** List of current programme instruments.

|  | Baseline | | Follow-Up | |
| --- | --- | --- | --- | --- |
|  | Cases | Controls | Cases | Controls |
| **Core** |  |  |  |  |
| Screening Schedule for Psychosis | ✓ | x | x | x |
| MRC Sociodemographic Schedule | ✓ | ✓ | ✓ | ✓ |
| Schedules for Clinical Assessment in Neuropsychiatry | ✓ | x | ✓ | x |
| Global Assessment of Function | ✓ | ✓ | ✓ | ✓ |
| WHO Disability Assessment Schedule | ✓ | ✓ | x | x |
| Personal and Psychiatric History Schedule (Baseline) | ✓ | x | x | x |
| Personal and Psychiatric History Schedule (Follow-Up) | x | x | ✓ | x |
| **Batch 1** |  |  |  |  |
| Premorbid Adjustment Scale | ✓ | ✓ | x | x |
| Family Interview for Genetic Studies | ✓ | ✓ | x | x |
| Childhood Experiences of Care and Abuse | ✓ | ✓ | x | x |
| Harvard Trauma Questionnaire | ✓ | ✓ | x | x |
| **Batch 2a** |  |  |  |  |
| List of Threatening Events | ✓ | ✓ | x | x |
| Family Interview Schedule | ✓ | ✓ | x | x |
| Significant Others Scale | ✓ | ✓ | x | x |
| **Batch 2b** |  |  |  |  |
| Neurological Evaluation Scale | ✓ | ✓ | x | x |
| [Obstetrics] | ✓ | ✓ | x | x |

In addition, we will explore the feasibility in each setting of collecting and processing DNA samples and conducting structural MRI scans.

**Training and Inter-Rater Reliability**

**Aim** To implement face to face and online training for researchers on all instruments and to evaluate inter-rater reliability, as appropriate

**Appendix 2.** Phase 1 INTREPID catchment areas.

**Chengalpet taluk, India**

Chengalpet taluk is a subdivision of the Kanchipuram district of Tamil Nadu, a state in the south east of India (see Figure 3). The town of Chengalpet is the administrative centre of the taluk and is around 50 kilometres south west of India’s fourth largest city and the state capital of Tamil Nadu, Chennai. The taluk is 1,130 km^2^, comprises 197 villages, and has a population of 412,289 according to the 2011 Indian census (population density: 365 per km^2^). The population aged 18 to 64 years is 259,742. According to the 2011 census, about half (54%) of the population of Chengalpet taluk lives in urban areas (i.e., areas with a population density of over 400 per km^2^). Traditionally, the economy of the taluk has centred on agriculture, animal husbandry, fishing and salt panning. This has begun to change on the back of wider economic shifts; in particular, close proximity to Chennai has contributed to the development of modern industries and services (e.g., automobile, engineering, service and information technology) particularly along the National Highway that links Chengalpet town with Chennai city.

**Ibadan South East and Ona Ara, Ibadan, Nigeria**

The city of Ibadan is the capital of Oyo State in south western Nigeria and is around 128 kilometres north east of Lagos (see Figure 4). Ibadan is 3,080 km^2^ and has a population of 2,338,659 according to the 2006 Nigerian census (population density: 759 per km^2^), making it the third largest city in Nigeria after Lagos and Kano. The catchment area for INTREPID comprises two of the eleven Local Government Areas (LGAs) in the Ibadan Metropolitan area: Ibadan South East (IBSE) (17 km^2^; population 266,457; population density: 15,673 per km^2^), which is located in the heart of the city; and Ona Ara (290 km^2^; population: 265,571; population density: 916 per km^2^), which is mainly rural. The population of the city is predominantly Yoruba (at least 90% or more of the population). Ibadan is a major trading centre and transit point between the coastal area in the south and the north of Nigeria, and the city’s economy is diverse. Its location means a major component of the city’s economy is trade (e.g., in cassava, cocoa, cotton, timber, rubber and palm oil) and there are many daily markets and small traders and artisans (e.g. gold smiths, mechanics, vulcanizers, tailors). In addition, the University of Ibadan (the oldest in Nigeria, with a student body of over 17,000) is a major source of employment; and, despite increasing urbanisation, farming and agriculture remain important.

**Tunapuna-Piarco, Trinidad**

The municipality of Tunapuna-Piarco is located in the north of Trinidad, just east of Port of Spain (see Figure 5), and covers an area of 522 km^2^, with a population of 248,656 according to the 2011 census (population density: 476 per km^2^). The low population density reflects the fact that there are large areas of barely populated bush, with the majority of the population living in a small number of more densely populated areas, mostly along the east-west corridor across the northern part of the island. The municipality is considered one of the better-off regions in Trinidad, with the exception of some of the more rural communities. It has the highest number of businesses in the country due to its favourable location between the capital and the east of Trinidad. Most of these businesses are situated on the Eastern Main Road (the east-west corridor), which runs from just east of Port of Spain to the east of the country. Trinidad is ethnically (East Indian, 37.0%; African, 31.8%; Mixed, 23.5%) and religiously (Roman Catholic, 22.3%; Hindus, 19.0%; and Pentecostal/Evangelical, 11.9) mixed and this is reflected in the population of Tunapuna-Piarco. The municipality has roughly equal numbers of African and of East Indian ethnicity, with a smaller proportion of mixed ethnicity; Roman Catholics are the largest religious group, followed by Hindus, and then Pentecostals. More broadly, despite its relative wealth, Trinidad faces a number of social problems. For example: Trinidad is a transhipment point for illegal drugs going to markets in North America and Europe, which has fuelled widespread drug use locally, it has the second highest homicide rate in the Caribbean and, much like its neighbours in the region, the country has relatively high rates of HIV/AIDS.

**Supplementary Table 1.** Chengalpet, India: Providers and Services for those with a Mental Disorder.†

a) Professional Sector

|  | | Provider | Location | Service | No. of beds | No. of users | Staff | Costs | Monitored |
| --- | --- | --- | --- | --- | --- | --- | --- | --- | --- |
|  | Chengalpet General Hospital | Public | In catchment area | Inpatient and daily outpatient clinics | 10 | 70 per clinic, approx. | 3 psychiatrists  2 nurses  1 ward assistant | None  (a family member is required to stay with the patient) | Yes |
|  | District Mental Health Programme  (Based at Chengalpet General Hospital; Medication dispensed in four primary health centres in Thiruporor Block of Chengalpet taluk) | Public | In catchment area | Outpatient clinics in local hospitals and health centres | - | 300 per week, approx. | 1 psychiatrist  1 psychologist  1 social worker | None | Yes |
|  | Schizophrenia Research Foundation (SCARF)  (Based in Chennai; Outreach clinics in catchment area) | NGO** | In catchment area | 3 outreach outpatient clinics per month | - | 30 per clinic, approx.  (all with psychoses) | 1 psychiatrist  1 social worker  5 health workers | None | Yes |
|  | Banyan | NGO** | In catchment area  (Kovalam) | Inpatient and 5 outpatient clinics per month | 12 | 50 per clinic, approx. | 1 psychiatrist (part-time)  1 coordinator  2 social workers  2 nurses  3 health workers | None | Yes |
|  | SRM Medical College* | Private | In catchment area | Inpatient | 13 | 13 | 1 professor of psychiatry  2 assistant professors  2 senior residents  4 nurses  2 support staff | Up to 30,000 rupees per week for admissions, assessments and medications  (approx. 480 US dollars) | Yes |
|  | Chettinad Medical College* | Private | In catchment area | Inpatient | 20 | 20 | 1 professor of psychiatry  1 assistant professors  2 senior residents  3 junior residents  4 nurses | Up to 30,000 rupees per week for admissions, assessments and medications  (approx. 480 US dollars) | Yes |
|  | Private psychiatrist 1 | Private | In catchment area  (Chengalpet Town) | Outpatient | - | 10 per day, approx. | 1 psychiatrist | Average cost of consultation is approx. 300 rupees  Average cost of medication for a month is approx. 300 rupees  (approx. 5 US dollars) | No |
|  | Private psychiatrist 2 | Private | In catchment area  (Chengalpet Town) | Outpatient | - | 10 per day, approx. | 1 psychiatrist | Average cost of consultation is approx. 300 rupees  Average cost of medication for a month is approx. 300 rupees  (approx. 5 US dollars) | Yes |
|  | Institute of Mental Health | Public | Outside catchment area  (Chennai City) | Inpatient and specialist daily outpatient clinics | 1,800 | 400 per clinic, approx. | Total 700 employees, including:  Psychiatrists at all levels  Psychologists  Nurses  Social workers | Consultation and treatment are free  Admission, cost based on the monthly income, with services free for those on less than 1000 rupees per week  (approx. 16 US dollars) | No |
|  | Psychiatric Unit, Madras Medical College* | Public | Outside catchment area  (Chennai City) | Inpatient and daily outpatient clinics | 12 | 150 per clinic, approx. | 1 professor of psychiatry  2 assistant professors  2 social workers  1 psychologist  5 nurses  2 hospital workers | None | No |
|  | Psychiatric Unit, Kilpauk Medical College | Public | Outside catchment area  (Chennai City) | Inpatient and daily outpatient clinics | 40  (occupancy approx. 50%) | 75 to 150 per clinic, approx. | 1 professor of psychiatry  1 assistant professor  2 senior residents  7 nurses  3 support staff | None  (a family member is required to stay with the patient) | No |
|  | Psychiatric Unit, Stanley Medical College | Public | Outside catchment area  (Chennai City) | Inpatient and daily outpatient clinics | 30  (mainly for detox.) | 100 per clinic, approx. | 2 professors of psychiatry  2 assistant professors  1 psychologist  2 social workers  5 nurses  3 support staff | Admission, nominal charge  (a family member or guardian is required to stay with the patient) | No |
|  | Schizophrenia Research Foundation (SCARF) | NGO** | Outside catchment area  (Chennai City) | Inpatient and daily outpatient clinics;  Tele-psychiatry | 150  (occupancy 100%) | 70 per clinic, approx. | 9 psychiatrists  1 psychologist  25 social workers  2 occupational therapists  3 nurses  5 psychiatry residents  25 administration and support staff | Admission, 350 rupees per day (2,450 rupees per week)  (approx. 6 US dollars per day [approx. 40 per week]) | Yes |
|  | Banyan | NGO** | Outside catchment area  (Chennai City) | Outreach services providing consultation and medication | - | 300 registered with psychoses, approx. | 1 psychiatrist  1 coordinator  6 social work student volunteers | None | No |
|  | Psychiatric Unit, Sri Ramachandra Medical College and Research Institute | Private | Outside catchment area  (Chennai City) | Inpatient and daily outpatient clinics | 70  (5 to 6 beds occupied at any one time) | 30 per clinic, approx. | 5 psychiatrists  6 psychologists  2 nurses  1 support worker | Admission, 15,000 to 25,000 rupees per week  (approx. 240 to 399 US dollars) | No |
|  | ACS Medical College and Hospital | Private | Outside catchment area  (Chennai City) | Inpatient and outpatient clinics | 0  (patients admitted to general beds) | 2 to 3 per week, approx. | 2 psychiatrists | None | No |
|  | Sree Balaji Medical College and Hospital | Private | Outside catchment area  (Chennai City) | Inpatient and outpatient | 30 | 1 to 2 per day, approx. | 3 psychiatrists  1 psychologist  4 nurses | Admission, 1,000 to 2,000 rupees per month  (approx. 16 to 32 US dollars) | No |
|  | Private psychiatrist A‡ | Private | Outside catchment area  (Chennai City) | Inpatient and outpatient | 0  (patients admitted to general beds) | 50 per day, approx. | 3 psychiatrists  1 psychologist  2 therapists  4 support staff | Admission, 1,000 to 3,000 rupees per day  (approx. 16 to 48 US dollars)  Consultation, 300 to 1,000 rupees  (approx. 5 to 16 US dollars) | Yes |
|  | Private psychiatrist B‡ | Private | Outside catchment area  (Chennai City) | Inpatient and outpatient | 0  (patients admitted to general beds) | 18 to 20 per day, approx. | 1 psychiatrist | Admission, 1,000 to 3,000 rupees per day  (approx. 16 to 48 US dollars)  Consultation, 300 to 1,000 rupees  (approx. 5 to 16 US dollars) | Yes |

† Information contained in the table is collated from various sources; figures relating to number of beds etc. should, consequently, be considered best estimates only

* A medical college is a training institution with an attached teaching hospital

** Non-governmental organisation

‡ There are around 120 psychiatrists who operate private practices in Chennai City. Of these, we identified 2 (A and B) who provide care (both inpatient in general beds when needed and outpatient) particularly for those with a psychotic disorder.

b) Folk Sector

|  | | Provider | Location | Service | No. of beds | No. of users | Staff | Costs | Monitored |
| --- | --- | --- | --- | --- | --- | --- | --- | --- | --- |
|  | Hanamanthapuram Temple  (Hindu) | - | In catchment area  (approx. 18 kilometres from Chengalpet town) | Residence in or near temple for up around 40 days†  Ritualised healing ceremonies | 8 to 10 individuals resident in or near temple at any point | 15 to 20 individuals visit per day  (this can increase to around 100 on auspicious days, e.g. new moon) | Temple Priests | None | Yes |
|  | Faith healers associated with Hanamanthapuram Temple  (i.e., healers who have set up practices in and around the temple)  (approx. 10 to 12) | - | In catchment area  (approx. 18 kilometres from Chengalpet town) | Rituals to counteract evil spells | - | Unknown  (one healer claimed to treat on average 6 to 8 individuals with a mental disorder per month) | Individual healers | No fixed charges | 1 healer |
|  | Dargah* at Kovalam  (Muslim**) | - | In catchment area  (approx. 40 kilometres from Chengalpet town) | Residence in or near Dargah for 48 days  Prayers and ritualised healing ceremonies | 10 to 12 individuals resident in or near Dargah at any point | 20 to 25 individuals visit per day | Individual healers | Consultation, 10,000 to 100,000 rupees  (approx. 160 to 1,610 US dollars)  Stay at Dargah and rituals, none | Yes |
|  | Muslim faith healers associated with Dargah at Kovalam  (i.e., healers who have set up practices in and around the temple)  (approx. 12 to 15) | - | In catchment area  (approx. 40 kilometres from Chengalpet town) | Prayers and ritualised healing | - | Unknown | Individual healers | No fixed charges | Yes |
|  | Pentecostal Church at Manamathy‡  (Congregation, approx. 100 families) | - | Manamathy | Prayers and staying at church for 1 to 2 days | Individuals stay at church during ‘treatment’ | 3 to 5 per month with mental disorder | Pastor | Donation to church, estimated average of 1,000 to 2,000 rupees  (approx. 2 to 4 US dollars) | Yes |

Note: Folk sector provision for those with a mental disorder in Chengalpet is faith based and delivered mainly by practitioners and healers located within churches or temples. At this stage, we were restricted to documenting the more prominent and larger churches and temples.

† Earlier, individuals who stayed at the temple were left chained by their families in the temple. This is now illegal and temple authorities do not allow it.

* Shrine of a Muslim saint

** Used by Muslims and Hindus

‡ There are a large number of Christian churches (approx. 200) of various denominations in Chengelpat taluk. We were not able to document all. We identified 6 of the larger churches. At only one of these (Pentecostal church at Manamathy) did the pastor say the church made specific provision for members of their congregation with mental health problems.

**Supplementary Table 2.** Ibadan South-East and On Ara, Nigeria: Providers and Services for those with a Mental Disorder.†

a) Professional Sector*

|  | | Provider | Location | Service | No. of beds | No. of users | Staff | Costs | Monitored |
| --- | --- | --- | --- | --- | --- | --- | --- | --- | --- |
|  | Adeoyo State Hospital | Public | Outside catchment area  (Ibadan South West) | Inpatient and outpatient clinics | 16 | 60 per clinic, approx. | 1 psychiatrist  1 senior medical officer  15 nurses | Admission free  Medication, around 1,000 to 2,000 naira pre month | Yes |
|  | University College Hospital | Public | Outside catchment area  (Ibadan North) | Inpatient and outpatient clinics | 62 | 100 per clinic, approx. | 10 psychiatrists  16 medical officers  22 nurses  2 social workers | Admission: deposit of 30,000 naira (covering 3 weeks of care); then around 1,600 naira per night (for bed and food)  (deposit: approx. 190 US dollars)  (per night: approx. 10 US dollars)  Medication, around 500 naira pre month  (approx. 3 US dollars)  Outpatient consultation, 1,250 naira  (approx. 8 US dollars) | Yes |
|  | New World Specialist Hospital | Private | Outside catchment area  (Ibadan South West) | Inpatient and outpatient clinics | 36 | 20 registered patients | 2 psychiatrists  1 medical officer  3 nurses | Patients pay for all services  Admission: unknown  Medication, 2,500 to 7,500 naira per month | Yes |

* There are no professional mental health services within the boundaries of Ibadan South East and Ona Ara.

b) Folk Sector*

|  | | Provider | Location | Service | No. of beds | No. of users | Staff | Costs | Monitored |
| --- | --- | --- | --- | --- | --- | --- | --- | --- | --- |
|  | Healer 1  (Pastor Ekeolu)  (Spiritual‡) | - | In catchment area  (Ibadan South East) | Residential**†  Prayer and other spiritual materials | 10 | 5, on average at any point | Healer, plus 2 others | Total, 25,000 naira and above depending on severity  (approx. 154 US dollars and above) | Yes |
|  | Healer 2  (Mrs Elijah)  (Spiritual‡) | - | In catchment area  (Ibadan South East) | Residential**†  Prayer and other spiritual materials | 10 | 5, on average at any point | Healer, plus 2 others | Total, 25,000 naira and above depending on severity  (approx. 154 US dollars and above) | Yes |
|  | Healer 3  (Prophet Salau)  (Spiritual‡) | - | In catchment area  (Ibadan South East) | Residential**†  Prayer and other spiritual materials | 5 | 1, on average at any point | Healer, plus 2 others | None | Yes |
|  | Healer 4  (Chief Mojeed)  (Traditional‡‡) | - | In catchment area  (Ibadan South East) | Residential**†  Herbs and sacrifices | 10 | 5, on average at any point | Healer, plus 2 others | Total, 20,000 to 30,000 naira  (approx. 123 to 184 US dollars) | Yes |
|  | Healer 5  (Prophetess Melody)  (Spiritual‡) | - | In catchment area  (Ibadan South East) | Residential**†  Prayer and water | 8 | 2, on average at any point | Healer, plus 2 others | Total, 20,000 to 40,000 naira  (approx. 123 to 246 US dollars) | Yes |
|  | Healer 6  (Prophet Johnson F)  (Spiritual‡) | - | In catchment area  (Ibadan South East) | Residential**†  Prayer and water | 8 | 2, on average at any point | Healer, plus 2 others | Total, 30,000 to 40,000 naira  (approx. 184 to 246 US dollars) | Yes |
|  | Healer 7  (‘Dr’ Adebayo)  (Spiritual‡) | - | In catchment area  (Ibadan South East) | Residential**†  Prayer and other spiritual materials | 6 | 1, on average at any point | Healer, plus 1 others | Total, 10,000 to 20,000 naira  (approx. 61 to 123 US dollars) | Yes |
|  | Healer 8  (Chief Dauda)  (Traditional‡‡) | - | In catchment area  (Ibadan South East) | Residential**†  Herbs and sacrifices | 12 | 5, on average at any point | Healer, plus 1 others | Total, 50,000 to 120,000 naira  (approx. 307 to 737 US dollars) | Yes |
|  | Healer 9  (Apena Olubajo)  (Traditional‡‡) | - | In catchment area  (Ibadan South East) | Residential**†  Herbs and sacrifices | 10 | 4, on average at any point | Healer, plus 2 others | Total, 30,000 naira and above depending on severity  (approx. 184 US dollars and above) | Yes |
|  | Healer 10  (‘Dr’ Bamijoko)  (Traditional‡‡) | - | In catchment area  (Ibadan South East) | Residential**†  Herbs and sacrifices | 8 | 5, on average at any point | Healer, plus 1 others | Total, 10,000 to 20,000 naira  (approx. 61 to 123 US dollars) | Yes |
|  | Healer 11  (‘Dr’ Olagunju)  (Traditional‡‡) | - | In catchment area  (Ibadan South East) | Residential**†  Herbs and sacrifices | 8 | 3, on average at any point | Healer, plus 1 others | Total, 5,000 to 10,000 naira  (approx. 31 to 61 US dollars) | Yes |
|  | Healer 12  (Alhaji Oloriosho)  (Traditional‡‡) | - | In catchment area  (Ibadan South East) | Residential**†  Herbs and sacrifices | 10 | 2, on average at any point | Healer, plus 2 others | Total, 20,000 naira and above depending on severity  (approx. 123 US dollars and above) | Yes |
|  | Healing Centre 1  (Emmanuel Rehabilitation Centre for Mentally Ill People)  (Spiritual‡) | - | In catchment area  (Ibadan South East) | Residential**†  (Many residents were chained)  Prayer  (The pastor claimed to be a qualified nurse and to give medication to residents) | 30 | 10, on average at any point | Healer, plus 7 others | 10,000 naira deposit for treatment  (approx. 61 US dollars)  5,000 naira deposit for food  (approx. 31 US dollars) | Yes |
|  | Healer 14  (Sheikh Amusa)  (Spiritual‡) | - | In catchment area  (Ona Ara) | Residential**†  Prayer, water and fasting | 10 | 5, on average at any point | Healer, plus 3 others | Total, 15,000 naira and above depending on severity  (approx. 92 US dollars and above) | Yes |
|  | Healer 15  (Prophet Omojola)  (Spiritual‡) | - | In catchment area  (Ona Ara) | Residential**†  Prayer and other spiritual materials | 10 | 3, on average at any point | Healer, plus 1 others | Total, 40,000 naira and above depending on severity  (approx. 246 US dollars and above) | Yes |
|  | Healer 16  (Imam Oyekola)  (Spiritual‡) | - | In catchment area  (Ona Ara) | Residential**†  Prayer and water | 5 | 2, on average at any point | Healer, plus 1 others | Total, 20,000 naira and above depending on severity  (approx. 123 US dollars and above) | Yes |
|  | Healer 17  (Chief Oladimeji)  (Spiritual‡) | - | In catchment area  (Ona Ara) | Residential**†  Prayer and water | 10 | 5, on average at any point | Healer, plus 2 others | Total, 15,000 naira and above depending on severity  (approx. 92 US dollars and above) | Yes |
|  | Healer 18  (Ogunse Olabode)  (Spiritual‡) | - | In catchment area  (Ona Ara) | Residential**†  Prayer and water | 15 | 5, on average at any point | Healer, plus 5 others | Total, 10,000 naira and above depending on severity  (approx. 61 US dollars and above) | Yes |
|  | Healer 19  (Ogunse Arididesi)  (Spiritual‡) | - | In catchment area  (Ona Ara) | Residential**†  Prayer, water and fasting | 15 | 4, on average at any point | Healer, plus 9 others | Total, 25,000 naira and above depending on severity  (approx. 154 US dollars and above) | Yes |

* We identified 19 of the more prominent healers (16 during the mapping phase; 3 during the case recruitment phase); 9 were identified through the Atorise Traditional and Faith Healers Association.

** We use ‘residential’ to denote that the healer provides accommodation for individuals during their ‘treatment’.

† During residential stays with healers, many individuals are chained to restrict movement (see Picture X).

‡ Spiritual healers rely primarily on faith based rituals to heal (e.g., prayer, fasting, baths, oil, soap, incense, and laying on of hands)

‡‡ Traditional healers incorporate use of herbs and sacrifices

**Supplementary Table 3.** Tunapuna-Piarco, Trinidad: Providers and Services for those with a Mental Disorder.†

a) Professional Sector

|  | | Provider | Location | Service | No. of beds | No. of users | Staff | Costs | Monitored |
| --- | --- | --- | --- | --- | --- | --- | --- | --- | --- |
|  | St Ann’s Hospital | Public | Outside catchment area  (St Ann’s)  (Although located outside the catchment area, it serves the catchment area) | Inpatient | 1,000 | 800 to 1,000 inpatients at any point | Sector 2 (Tunapuna-Piarco) Team at St Ann’s:  1 consultant psychiatrist  1 registrar  2 house officers  5 mental health officers  4 social workers | None | Yes |
|  | Eric Wiliams Medical Sciences Complex | Public | In catchment area | Inpatient and 2 outpatient clinics, per week | 0  (Patients admitted to general beds) | 50 per outpatient clinic, approx. | 3 consultant psychiatrists  2 registrars  4 house officers | None | Yes |
|  | Tacaragua Health Centre  (Tunapuna-Piarco Sector Mental Health Team) | Public | In catchment area | Outpatient clinic, weekly | - | 150 to 200 active patients, approx. | Sector 2 (Tunapuna-Piarco) Team at St Ann’s:  As for St Ann’s above | None | Yes |
|  | Arima Health Centre  (Tunapuna-Piarco Sector Mental Health Team) | Public | In catchment area | Outpatient clinic, every two weeks | - | 80 to 120 active patients, approx. | Sector 2 (Tunapuna-Piarco) Team at St Ann’s:  As for St Ann’s above | None | Yes |
|  | Prison In-reach Service  (Serving 4 prisons) | Public | In catchment area  (All prisons located in Arouca, in the east of the catchment area) | In-reach | - | 30 per week, approx. | 1 forensic psychiatrist  1 registrar | None | Yes |
|  | St Augustine Hospital | Private | In catchment area | Inpatient and consult. by appt. | 0  (Patients admitted to general beds) | 14 per week, approx. | 2 consultant psychiatrists | 400 to 700 Trinidad and Tobago dollars per appointment  (approx. 62 to 109 US dollars) | Yes |

b) Folk Sector*

|  | | Provider | Location | Service | No. of beds | No. of users | Staff | Costs | Monitored |
| --- | --- | --- | --- | --- | --- | --- | --- | --- | --- |
|  | Pastor  (Jesus the Light of the World Ministries)  (Pentecostal)  Spiritual Healer | - | In catchment area  (Heights of Guanapo) | Prayer  (To heal those disturbed by demons) | - | Unknown | Pastor | Unknown | Yes |
|  | Kali Priest  (Kali Matta Devi Mandir)  (Hindu, Kali)  Spiritual Healer | - | In catchment area  (Tunapuna) | Prayer, exorcism, spirit removal  (To heal victims of demonic possession; if aggressive, have to be tied down until spirit removed) | - | Unknown | Kali Priest, plus one other | Unknown | Yes |
|  | Pastor  (Solomon Temple Church)  (Pentecostal)  Spiritual Healer | - | In catchment area  (Wallerfield) | Prayer, exorcism  (To heal victims of demonic possession) | - | 50, approx. over unknown time period | Pastor | None | Yes |
|  | Pastor  (Prayer and Deliverance Temple Ministries)  (Pentecostal)  Spiritual Healer | - | In catchment area  (Carapo, Arima) | Prayer, exorcism  (To heal victims of demonic possession) | - | Unknown | Pastor | Unknown | Yes |
|  | Reverend Mother  (St Anthony’s Spiritual Baptist)  (Oshun Shrine)  Traditional, Spiritual Healer | - | In catchment area  (St Helena) | Prayer, cleansing, herbal remedies, purifying rituals, spirit removal  (Some stay at shrine for a few months until healed) | Unknown; some people stay if needed | Unknown | Reverend Mother | Total, $100 + Trinidad and Tobago dollars  (approx. 16 + US dollars) | Yes |
|  | Catholic Charismatic Renewal Minister  (Eternal Light Communities)  Spiritual Healer | - | In catchment area  (Tunapuna) | Spirit removal, removal of obeah, healing from physical and mental afflictions, counselling | - | 10 per meeting | 3 individuals involved in prayers, healing and guidance | Unknown | Yes |
|  | Pastor  (D’Abadie Pentecostal Church)  Spiritual Healer | - | In catchment area  (D’Abadie) | Healing, Prayer, exorcism, removal of spirits  (To heal spiritual problems, possession) | - | Unknown | Pastor | Unknown | Yes |
|  | SDA Pastor  (The South Caribbean Conference of Seventh-day Adventists)  Spiritual Healer | - | In catchment area  (St Augustine) | Prayer, exorcism  (To heal spirit possession) | - | Unknown | Pastor | Unknown | Yes |
|  | Pastor  (Divine Encounter Fellowship Ministries International)  Spiritual Healer | - | In catchment area  (D’Abadie) | Exorcism, removal of spirits, healing of spiritual problems | - | Unknown  (large congregation; many will present with problems at services) | Pastor | Unknown | Yes |
|  | Pentecostal Pastor  (New Testament Church of God)  Spiritual Healer | - | In catchment area  Tunapuna | Healing spiritual problems, spirit removal | - | Unknown | Pastor | Unknown | Yes |
|  | Catholic Priest  (Church of the Incarnation)  Spiritual Healer | - | In catchment area  (Arima) | Healing mass, spirit removal  (also holds a healing mass at Our Lady of All Nations Church, still within the sector) | - | Unknown | Priest | Unknown | Yes |
|  | Bishop  (Arouca Revival Tabernacle)  Spiritual Healer | - | In catchment area  (Arouca) | Removal of spirit possession and other physical, spiritual, mental afflictions | - | Unknown | Bishop | Unknown | Yes |
|  | Kali Priest  (Sri Govinda Maha Kali Mandir)  Spiritual Healer | - | In catchment area  (Tunapuna) | Prayer, exorcism  (To heal victims of demonic possession; | - | Unknown | Kali Priest | Unknown | No |
|  | Pastor  (Faith Revival Ministries World Outreach)  Spiritual Healer | - | In catchment area  (Arima) | Casting out demons; healing by fasting, prayer, deep faith, and the laying on of hands | - | Unknown  (Encounters many people with problems; large congregation) | Pastor, plus one other | Unknown | No |
|  | Bush Doctor 1  Traditional Healer | - | In catchment area  (Blanchissues) | Herbs and spiritual rites | - | Only occasionally treats those with mental disorders | Bush doctor | Unknown | No |
|  | Spiritualist and Herbalist | - | In catchment area  (Heights of Guanapo) | Herbs to heal physical and spiritual problems | - | Only occasionally treats those with mental disorders | Unknown | Unknown | No |
|  | Bush Doctor 2 | - | In catchment area  (Peytonville) | Unknown | - | Unknown | Bush doctor | Unknown | No |
|  | Spiritual Healer  (St Anthony’s House of Prayer Light) | - | In catchment area  (Champ Fluer) | Prayer; spiritual counselling; fasting; offerings; spiritual baths; herbal medication; other rites and rituals | - | Unknown | Healer | Unknown | No |
|  | (Pastor)  Piarco Pentecostal Church | - | In catchment area  (Piarco) | Unknown | - | Unknown | Pastor | Unknown | No |
|  | Pastor  (La Horquetta Gospel Foundation) | - | In catchment area  (La Horquetta) | Unknown | - | Unknown | Pastor (a retired psychiatric nurse) | Unknown | No |
|  | Healer  (Mama Oshun African Shrine) | - | In catchment area  (St Augustine) | Herbs, prayers, spiritual rites, cleansing | - | Unknown | Healer | Unknown | No |
|  | Pastor  (Precious Blood Community) | - | In catchment area  (Arima) | Charismatic prayer group | - | Rarely | Pastor | Unknown | No |
|  | Abbot  (Mt St Benedict) | - | In catchment area  (Tunapuna) | Prayer, blessings | - | Unknown | Abbot | None | No |

* The folk sector in Trinidad is diverse and disparate, reflecting its cultural and religious diversity. Healing is mainly delivered by pastors and church leaders to their congregations. Many provide healing for individuals suffering from a mental disorder, but none (as far as we could ascertain) specialised in mental disorders. We identified 24 [23] healers.
